# Allosteric activation of preformed EGF receptor dimers by a single ligand binding event

**DOI:** 10.3389/fendo.2022.1042787

**Published:** 2022-11-30

**Authors:** Endang R. Purba, Ei-ichiro Saita, Reetesh R. Akhouri, Lars-Goran Öfverstedt, Gunnar Wilken, Ulf Skoglund, Ichiro N. Maruyama

**Affiliations:** ^1^ Information Processing Biology Unit, Okinawa Institute of Science and Technology Graduate University, Okinawa, Japan; ^2^ Cellular Structural Biology Unit, Okinawa Institute of Science and Technology Graduate University, Okinawa, Japan

**Keywords:** cancer biology, conformational change, cooperativity, cryo-electron tomography, receptor tyrosine kinase, signal transduction, single-molecule biophysics, transmembrane signaling

## Abstract

Aberrant activation of the epidermal growth factor receptor (EGFR) by mutations has been implicated in a variety of human cancers. Elucidation of the structure of the full-length receptor is essential to understand the molecular mechanisms underlying its activation. Unlike previously anticipated, here, we report that purified full-length EGFR adopts a homodimeric form *in vitro* before and after ligand binding. Cryo-electron tomography analysis of the purified receptor also showed that the extracellular domains of the receptor dimer, which are conformationally flexible before activation, are stabilized by ligand binding. This conformational flexibility stabilization most likely accompanies rotation of the entire extracellular domain and the transmembrane domain, resulting in dissociation of the intracellular kinase dimer and, thus, rearranging it into an active form. Consistently, mutations of amino acid residues at the interface of the symmetric inactive kinase dimer spontaneously activate the receptor *in vivo*. Optical observation also indicated that binding of only one ligand activates the receptor dimer on the cell surface. Our results suggest how oncogenic mutations spontaneously activate the receptor and shed light on the development of novel cancer therapies.

## Introduction

The human epidermal growth factor receptor (EGFR) family, a member of the receptor tyrosine kinase (RTK) superfamily, plays vital roles in various cellular processes, including cell survival, proliferation, differentiation, motility, and metabolism (1, 2). The EGFR signaling pathway is one of the most dysregulated pathways in many human cancers (3, 4). The EGFR family members, EGFR (also known as ErbB1 and HER1), ErbB2/HER2/Neu, ErbB3/HER3 and ErbB4/HER4, are all synthesized as type-1 single-pass transmembrane proteins. Seven known ligands, such as epidermal growth factor (EGF) and transforming growth factor-α, activate EGFR, while neuregulins (NRGs) bind to ErbB3 and ErbB4 (5). ErbB2 is an orphan receptor, for which a peptide ligand has not been found, although it can be activated by mildly alkaline pH (6) or by forming heterodimers with other family members (7).

**Figure 1:**
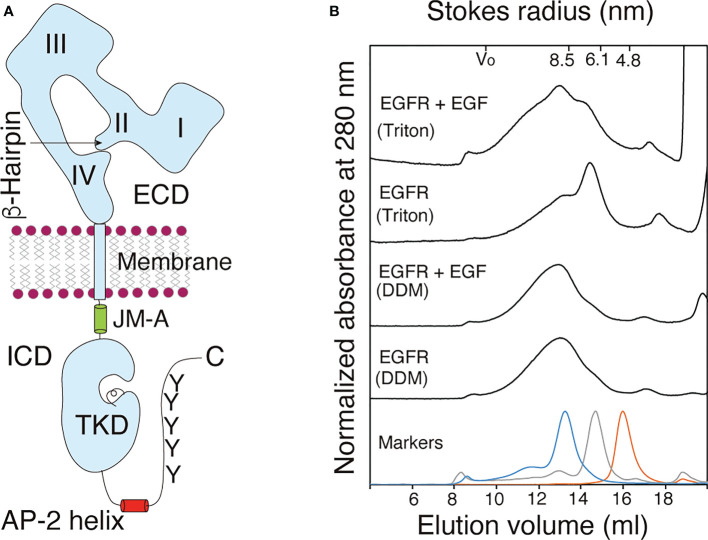
Purified EGFR adopts a dimeric structure before and after ligand binding. **(A)** Schematic representation of an EGFR monomer. The extracellular domain (ECD) of EGFR consists of the four subdomains I-IV. The β-hairpin of the subdomain II interact with the tethering arm of the subdomain IV for the receptor's closed (tethered) form. The intracellular domain (ICD) of the receptor comprises intracellular juxtamembrane (JM), the tyrosine kinase domain (TKD), AP-2 helix and tyrosine (Y) residues as major phosphorylation sites among others. Not drawn to scale. **(B)** Gel filtration chromatograms of purified full-length EGFR solubilized from the membrane by using 1.0% DDM (DDM) or 1.0% Triton X-100 (Triton) before (EGFR) and after (EGFR+EGF) ligand binding. Elution patterns of molecular mass markers are shown at the bottom. A representative of three chromatograms independently carried out.

The EGFR protein, ~170 kDa in mass, consists of an extracellular ligand-binding domain, single transmembrane domain (TMD), intracellular juxtamembrane (JM) region, cytoplasmic tyrosine kinase domain (TKD), and C-terminal tail (**Figure 1A**) (8). Crystallographic studies of the isolated extracellular domain (ECD) and intracellular domain (ICD) of EGFR have provided insight into liganded and unliganded forms of the receptor. The ECD contains four subdomains (9–11). Subdomains I (also known as L1) and III (L2) have a β-helix solenoid structure and are responsible for ligand binding by simultaneously contacting the same ligand bound. Subdomains II (CR1) and IV (CR2) are cysteine-rich and interact with each other in the unliganded, tethered form. Ligand binding to subdomains I and III breaks this intramolecular contact for intermolecular association of two β-hairpins of subdomain II in its liganded, extended form. Crystal structures of symmetric and asymmetric TKD dimers have also been determined as the inactive and active forms of the receptor, respectively (12–16).

The traditional model of RTK activation is that a ligand binds to the monomeric receptor and induces receptor dimerization. This brings the intracellular TKDs into close proximity, resulting in kinase activation and phosphorylation of the receptor and its substrate tyrosine residues (17). This model was first proposed for the EGFR (18), and phosphotyrosines of the receptor interact with effector molecules, including the Src homology 2 domain-containing transforming protein-1 (Shc1) and growth factor receptor-bound protein-2 (Grb2) adapters, for downstream signaling (19–21). Consistent with this model, loose linkage between ligand binding and kinase activation of the receptor has been proposed (22). Furthermore, a nearly full-length EGFR protein was purified as a monomer, which upon ligand binding, became a dimer *in vitro* (23). When full-length EGFR was ectopically expressed at low levels in *Xenopus* oocytes, the receptor was predominantly monomeric in the absence of ligand, and the addition of EGF generated dimers and oligomers (24).

Concave-up curvilinear Scatchard plots were first described for the interaction between EGF and its cell surface receptor (25, 26), and has traditionally been interpreted as heterologous, high-affinity and low-affinity, sites on the cell surface, which correspond to dimeric and monomeric receptors, respectively (27). However, Macdonald and Pike (28) have recently argued that the concave-up Scatchard plots arise from negative cooperative EGF binding to the preformed EGFR dimer, as previously predicted (29). Indeed, numerous biochemical and optical imaging studies have demonstrated that in the absence of bound ligand, EGFR adopts a dimeric, yet inactive, form at various levels on the cell surface, depending on methods and cell lines used for the analysis (30–37).

There are a number of oncogenic mutations that spontaneously activate EGFR in the absence of bound ligands (38–40). Among such mutations, deletion mutations in the receptor’s ICD were found. EGFRvIVa lacks three exons 25-27, resulting in a C-terminal deletion of residues 959-1066 (41–43). EGFRvIVb lacks two exons 25 and 26, resulting in a C-terminal deletion of residues 959-1030 (41–43). These tumorigenic mutations suggest that EGFR is actively inhibited prior to ligand binding (44, 45). However, the ligand-induced dimerization model does not explain the tumorigenic activity of these EGFR mutants.

In the present study, we analyzed the structures of full-length EGFR in the absence and presence of a bound ligand. When purified EGFR was analyzed by gel filtration chromatography, it behaved as a dimer before and after activation upon ligand binding. Cryo-electron tomography (Cryo-ET) analysis of the purified, full-length receptor also showed dimeric unliganded and liganded receptors, the latter of which showed a relatively stable ECD structure with a bound ligand. Consistent with these *in vitro* results, artificial mutations of amino acid residues at the interface of the symmetric inactive kinase dimer spontaneously activate the receptor *in vivo*. Optical observation also showed that binding of only one ligand activates the receptor dimer on the cell surface. Furthermore, ligand-induced phosphorylation was essential for dimerization and oligomerization of the receptor dimers.

## Results

### EGFR adopts a dimeric structure *in vitro*

Full-length human EGFR tagged with eight histidine residues (His tag) at its C-terminus was expressed in human embryonic kidney HEK293T cells and was purified by nickel chelating Sepharose column chromatography after solubilization with 1.0% (w/v) n-dodecyl-β-D-maltoside (DDM) (**Supplementary Figure S1**). The purified full-length EGFR was observed to be phosphorylated at the basal level when analyzed by immunostaining with an anti-phosphotyrosine antibody. Upon stimulation with its ligand, epidermal growth factor (EGF), autophosphorylation of full-length EGFR was enhanced markedly *in vitro* in the presence of ATP (**Supplementary Figure S2**), indicating that the purified receptor molecules are functional.

When analyzed by gel filtration chromatography, the full-length receptor molecules solubilized with DDM were eluted as a symmetric peak with an average Stokes radius (± SD) of 9.05 ± 0.34 nm or 9.01 ± 0.46 nm before or after incubation with EGF, respectively (**Figure 1B**). In contrast, full-length EGFR receptor molecules solubilized with 1.0% Triton X-100 were eluted as two peaks with the Stokes radii of 6.96 ± 1.03 nm and 8.95 ± 0.73 nm (**Figure 1B**). The lower molecular mass peak shifted to the position of the higher peak after incubation with EGF, indicating that monomeric EGFR became a dimer upon ligand binding. These results indicated that the full-length EGFR molecules with and without bound EGF adopt homodimeric structures when solubilized with 1.0% DDM. When solubilized with 1.0% Triton X-100, in contrast, a large fraction of the full-length EGFR adopted a monomeric structure, which upon EGF binding, became dimeric. When an EGFR mutant that lacks its C-terminal tail was solubilized with 0.2% Triton X-100, the mutant receptor was completely monomeric (46), indicating that the C-terminal tail stabilizes its dimeric structure. The longer hydrophobic tail and/or smaller hydrophilic headgroup of Triton X-100 than DDM may destabilize the receptor’s dimeric structure. To exclude the possibility that the His tag contributes to the formation of the dimeric structure, the tag was cleaved from the full-length EGFR by digestion with tobacco etch virus (TEV) endopeptidase (**Supplementary Figure S3**). The Stokes radius of the cleaved EGFR was similar to that of the full-length EGFR with the His tag, indicating that the tag does not contribute to dimer formation. These results show that prior to ligand binding, full-length EGFR has a homodimeric structure and that the receptor dimer can be activated by ligand binding without changing its dimeric form.

### 3D density maps of purified EGFR

**Figure 2:**
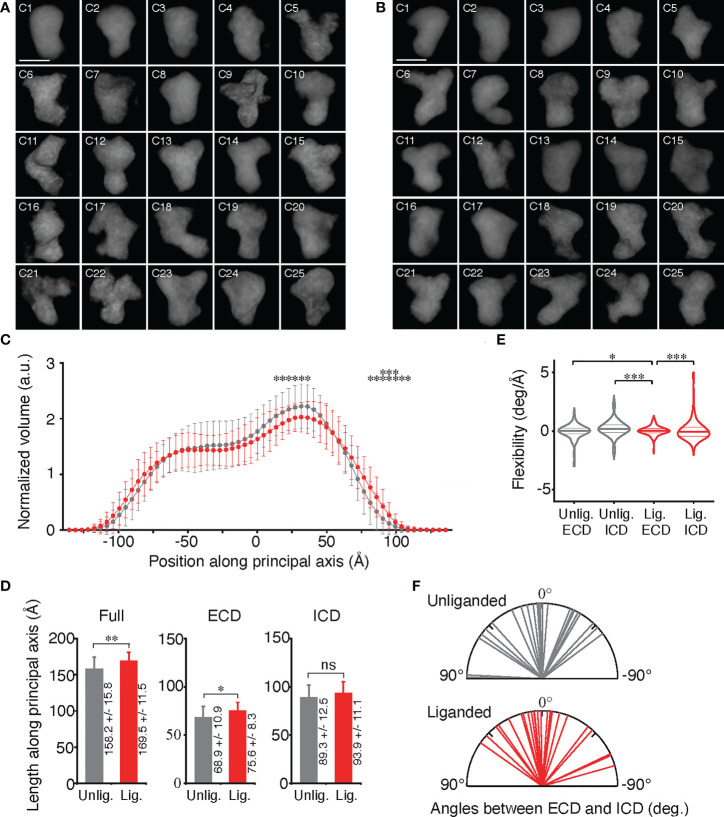
Conformational variables of EGFR before and after activation. **(A, B)** Averaged electron density maps of 25 subtomogram clusters of unliganded and liganded EGFRs, respectively. **(C)** Plots of averaged volumes of unliganded (gray) and liganded (red) EGFRs along the principal axis with distances from the molecular center of mass. Data points are mean ± SD. Two-sided Student's *t*-test in the range between -140Å and 75Å or Mann-Whitney U test in the range over 80Å along the principal axis (*p < 0.05, **p < 0.01). **(D)** Long-axis lenghts of ECD and ICD of unliganded and liganded EGFRs were measured as shown in **Supplementary Figure S8A**. Data points are mean ± SD. Two-sided Student's *t*-test. ns, not significant. **(E)** Flexibility of ECD and ICD of unliganded and liganded EGFRs, which was measured as shown in **Supplementary Figure S8B**. Lines in the violin plots show 25^th^, 50^th^ and 75^th^ percentile values. Asterisks indicate that variances are significantly different (Levene's test; *p < 0.05, ***p < 0.001). **(F)** Angles between long axes of ECD and ICD of unliganded and liganded EGFRs, which were measured as described in **Supplementary Figure S8A**.

We collected 18 and 15 tomograms by cryo-electron tomography (Cryo-ET) of the purified full-length EGFR without and with bound EGF, respectively (**Supplementary Table S1**). Cryo-ET is more sensitive than single particle cryo-electron microscopy to detect various conformers of EGFR before and after ligand binding. After refinement using the constrained maximum entropy tomography (COMET) software package (47), final three-dimensional (3D) “density maps” of the proteins were calculated from forward-scattered electrons at 300 kV. Within these tomograms (**Supplementary Figure S4**), the MINER program of the package was used to extract subtomograms. We reconstructed 474 molecules of full-length unliganded EGFR from the 18 tomograms. The CORRPAIR program was applied to produce correlation matrices between pairs of all the subtomograms and to classify the 474 subtomograms into 25 clusters (**Supplementary Figure S5**). Using the CORRAVE program, subtomograms of each cluster were averaged to represent the respective cluster (**Figure 2A**). From the 15 tomograms, we also reconstructed 557 liganded EGFR molecules activated by EGF binding, which were classified into 25 clusters (**Supplementary Figure S6**). Subtomograms of each cluster were averaged, as shown in **Figure 2B**.

The 25 averaged density maps of unliganded or liganded EGFRs were analyzed by determining the principal axis of minimum moment of inertia and the molecular center of mass. The mean volumes of slices of both the unliganded and liganded receptors along the principal axis showed two peaks, one of which was larger than the other (**Figure 2C**). Based on the crystal structures of EGFR ECD and ICD, the results indicated that the large and small peaks correspond to ECD and ICD dimers, respectively, which are separated by the TMDs (**Supplementary Figure S7**). Furthermore, full lengths and ECD lengths of the liganded receptors were significantly longer than those of the unliganded receptors (**Figure 2D**), suggesting that a fraction of the unliganded ECD may take a tethered structure through the interaction of the subdomains II and IV, whereas liganded EGFR ECD may have an extended structure (9–11).

When the flexibility of ECD and ICD of unliganded and liganded EGFRs was examined (**Supplementary Figures S8A, B**), it was observed that the ECD dimer was significantly stabilized by ligand binding and had the most rigid structure (**Figure 2E**). Angles between the long axes, which are perpendicular to the principal axis of minimum moment of inertia, of ICD and ECD of the unliganded EGFRs were variable and ranged from 89° (clockwise) to –54° (counterclockwise) when observing the molecules extracellularly (**Figure 2F**; **Supplementary Figures S9D, E**). Similarly, angles between the long axes of ICD and ECD of the liganded EGFRs also ranged from 55° to –74°. These rotation angle variabilities were not significantly different between the unliganded and liganded receptors, indicating that the receptor dimers twist flexibly perpendicular to the principal axis before and after ligand binding.

### Conformational flexibility transition

**Figure 3:**
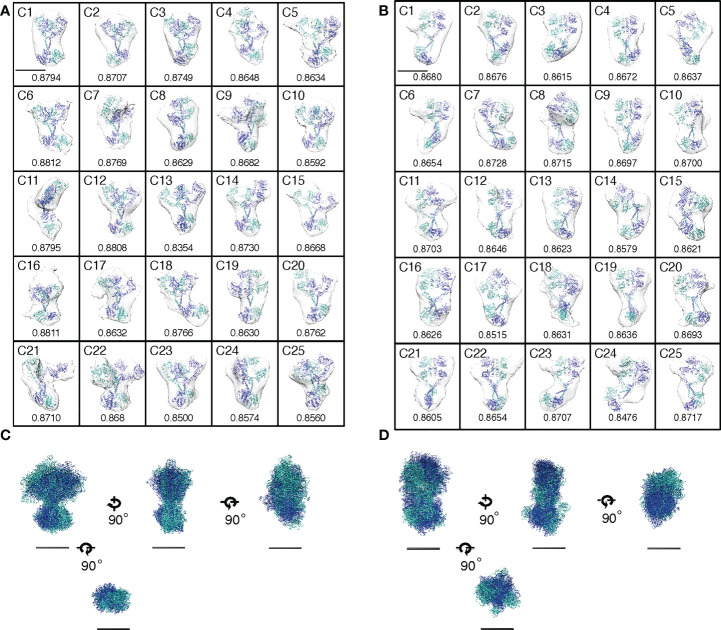
Activation of EGFR dimers by conformational flexibility transition or by mutations. **(A, B)** Envelopes of averaged density maps of unliganded and liganded EGFRs, respectively, were docked with crystal and NMR structures. Cross-correlation coefficients between the averaged density maps and the crystal/NMR structures are shown below each map. Scale bar, 10 nm. **(C, D)** Alignments of crystal and NMR structures docked into the averaged density maps of unliganded or liganded EGFR, respectively, along two principal axes of minimum and maximum moments of inertia and the molecular center of mass. Four orthogonal views are shown, but ECDs are removed from bottom views for clarity. Scale bar, 10 nm.

Density maps from the 3D reconstruction were sufficient for defining ECD and ICD and their conformational changes, as described above. Using CHIMERA software (48), crystal structures of EGFR domains were manually docked into the envelope of each averaged subtomogram of 25 unliganded and 25 liganded receptor clusters (**Figure 3**). Crystal structures of the tethered (PDB ID: 1NQL) or extended (half of 3NJP) ECD monomer were docked into the envelope of unliganded receptors. The ECD dimer (3NJP) was docked into the envelope of liganded EGFRs. The crystal structure (3GT8) of a symmetric inactive TKD dimer and the NMR structure (2M0B) of inactive TMDs were docked into the envelope of unliganded EGFRs (**Figure 3A**), whereas the crystal structure (2GS6) of the asymmetric active TKD dimer and the NMR structure (2M20) of active TMDs were docked into the envelopes of liganded receptors (**Figure 3B**).

Then, the 25 docked crystal structures of unliganded or liganded receptors were manually aligned to each other along two principal axes of minimum and maximum moments of inertia and the molecular center of mass (**Figures 3C, D**). These alignments show that both ECD and ICD dimers of unliganded and liganded EGFR dimers have flexible structures, which are likely to correspond to large rotation angles between ECD and ICD of the averaged density maps, as shown in **Figure 2F**. The spontaneous structural transition of unliganded receptor ECD from tethered to extended is also likely to contribute to the flexibility of the domain (49, 50). Such a spontaneous transition was predicted in the wild-type receptor by molecular dynamics analysis (51). Furthermore, an NMR study of EGFR in native membranes has also shown that ECD of the unliganded receptor is highly dynamic, while ICD is rigid (52). This relative stability of ICD is consistent with its role in the formation of the unliganded receptor dimer, as described below. In contrast, ECD dimers of the liganded receptor showed the most rigid structures among other domains of unliganded and liganded receptors (**Figure 2E**), consistent with the previous small angle X-ray scattering study of the ECD (50). Although our flexibility analysis (**Figure 2E**) did not detect statistically significant flexibility transition in the ICDs of the EGFR dimer, it may also be true that the ICDs become flexible upon ligand binding (compare **Figures 3C, D**). Upon ligand binding, therefore, conformational flexibility transition occurs in ECDs of the EGFR dimer from a flexible to rigid structure, and flexibility transition may also occur in ICDs of the receptor dimer from a rigid to flexible structure.

### Role of ICD in preformed dimers *in vivo*

**Figure 4:**
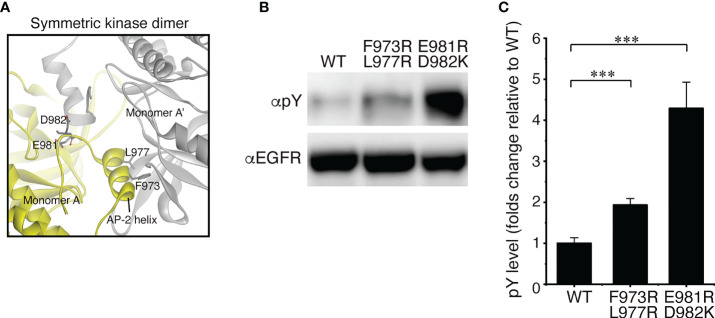
EGFR mutations that spontaneously activate the receptor. **(A)** Crystal interface between protomers of symmetricTKD dimer, each of which is shown in gray or yellow. **(B)** Western blots of wild-type EGFR and two mutants with two amino acid substitutions. **(C)** Summary of five independent Western blots. Data points are means ± SEM. Two-sided Student's *t*-test (***p < 0.001).

As described above, our structural analysis of the solubilized EGFR with DDM showed that the receptor is dimeric *in vitro*. Therefore, we also examined whether EGFR adopts a homodimeric form *in vivo*. Full-length EGFR was expressed in modified HeLa cells that did not express any of the four EGFR family members on the cell surface to prevent heterodimerization with endogenous receptors (**Supplementary Figures S10A-C**). A structural study indicates that a symmetric inactive TKD dimer is stabilized by the AP-2 helices, which interact with the interfaces of two protomers of the dimer (16) (**Figures 1A** and **4A**). The “electrostatic hook”, which consists of acidic residues in the turn after the AP-2 helix, also forms ion pairs with residues in the other subunit. When we mutated Phe-973 and Leu-977 of the AP-2 helix, which forms hydrophobic interactions with residues of the other protomer, to hydrophilic arginine residues, the mutant receptor was spontaneously autophosphorylated in the absence of bound ligand (**Figures 4B, C**). When negatively charged Glu-981 and Asp-982 of the “electrostatic hook” were mutated to positively charged arginine and lysine residues, respectively, the mutant receptor was also spontaneously activated. Consistently, a substitution of four negatively charged amino acid residues in the “electrostatic hook” makes the mutant receptor spontaneously active (53). These results indicate that the mutations destabilize the kinase dimer for spontaneous activation, suggesting that the C-terminal tail encompassing the AP-2 helix and “electrostatic hook” plays a major role in the formation of unliganded dimers (32, 37).

### Single ligand activates EGFR *in vivo*

**Figure 5:**
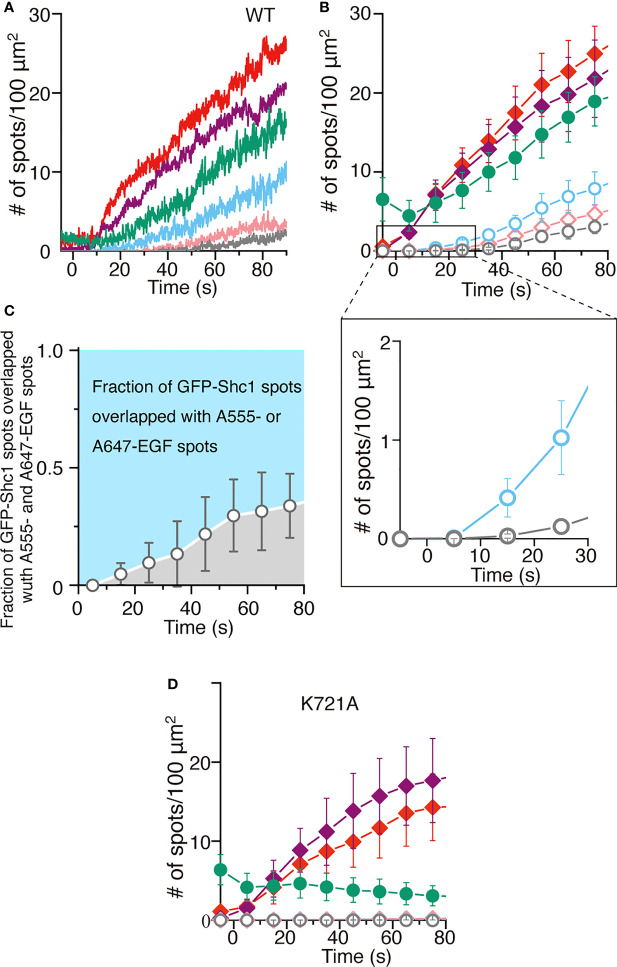
Single ligand binding activates EGFR dimers. **(A)** Time courses of the number of spots of Alexa555-labeled EGF (red), Alexa647-labeled EGF (purple) or GFP-Shc1 (green) appeared on the surface of modified HeLa cells exogenously expressing EGFR. Colocalization of GFP-Shc1 and either Alexa555-EGF or Alexa647-EGF, of Alexa555-EGF and Alexa647-EGF, or of all the three is shown in light blue, pink and gray, respectively. The fluorescently labeled EGF molecules, 0.1 nM each at a final concentration, were incubated with cells expressing EGFR. **(B)** Same as in **(A)**. Data points are means ± SEM (n=4). **(C)** The fraction of GFP-Shc1 spots overlapped with both Alexa555- and Alexa647- or Alexa-647-EGF spots. **(D)** Time courses of the number of fluorescent spots as in **(A)**, which appeared on the surface of cells exogenously expressing kinase-dead (k721A) EGFR. Data points are means ± SEM (*n*=4).

The homodimeric structure of EGFR described above is contradict with the ligand-induced dimerization model proposed for the activation of EGFR. The model predicts that a ligand binds to the monomeric receptor and induces receptor dimerization (18). Using the highly inclined and laminated optical sheet (HILO) illumination (54), therefore, we tried to optically observe the EGFR activation on the top surface of living cells in real time. Full-length human EGFR was expressed exogenously in the modified HeLa cells (**Supplementary Figure S10**) that do not express any of the four EGFR family members on the cell surface to prevent spontaneous heterodimerization (37). To monitor the activation of the receptor by ligand binding *in vivo*, we also expressed the Shc1 adaptor protein fused with GFP (GFP-Shc1) in the modified HeLa cell, which upon the receptor phosphorylation, is recruited to the cell surface by interacting with the receptor’s phosphortyrosine residues. When two fluorescently labeled EGF molecules, 0.1 nM (~0.6 ng/ml) each of Alexa555-EGF and Alexa647-EGF at a final concentration, were simultaneously incubated with the cell culture, the number of EGF fluorescence spots (red or purple) was gradually increased during the incubation time (**Figures 5A, B**). Each spot seems to initially represent a single EGF molecule since the spot appeared in a single step within a few video frames (**Supplementary Video S1**). To support this, furthermore, colocalization of Alexa555-EGF (red) and Alexa647-EGF (purple) spots could not be detected at the beginning of the incubation (**Figures 5A, B**).

The number of EGF fluorescence spots was more than twice of that of GFP-Shc1. This suggests the following two possibilities: (1) EGF binding does not always activate the EGFR molecule. (2) A significant amount of endogenous, unlabeled Shc1 interacts with activated EGFR and prevent GFP-Shc1 binding to the activated receptor. The number of GFP-Shc1 (green) was always larger than that of colocalization (light blue) of GFP-Shc1 and either Alexa555- or Alexa647-EGF, suggesting that the fluorescently labeled EGF solution contains unlabeled and/or photo-bleached EGF molecules. Colocalization of GFP-Shc1 and Alexa555- or Alexa647-EGF spots (light blue in **Figures 5A, B**) was much faster than that of Alexa555- and Alexa647-EGF spots (pink) or of GFP-Shc1 and Alexa555- and Alexa647-EGF spots (gray) (**Figures 5A, B**). Similar results were obtained by expressing Grb2 fused with GFP (Grb2-GFP) in the modified HeLa cell (**Supplementary Figures S11B, C**). When colocalization of GFP-Shc1 (green) and either Alexa555- or Alexa647-EGF (light blue) or of Alexa555- and Alexa647-EGF (gray) was analyzed (**Figure 5C**), only Alexa555- or Alexa647-EGF initially colocalized with GFP-Shc1, followed by colocalization of GFP-Shc1 and Alexa555- and Alexa647-EGF at later stages. Furthermore, the majority of activated EGFR molecules interacts with single EGF molecules (**Figure 5C**).

Interestingly, colocalization of Alexa555- and Alexa647-EGF spots and of GFP-Shc1 and Alexa555- and Alexa647-EGF spots was not observed when kinase-dead EGFR was expressed on the cell surface (**Figure 5D**), or the kinase was inhibited by a specific inhibitor (**Supplementary Figures S11D, E**). These results indicate that singly liganded EGFR autophosphorylates the receptor without interacting with another singly liganded receptor. This is inconsistent with the ligand-induced dimerization model, where two liganded monomers interact with each other to autophosphorylate in *trans*. Therefore, the simplest explanation of the results is that the colocalization of Alexa555- and Alexa647-EGF or of GFP-Shc1 and Alexa555- and Alexa647-EGF spots is due to dimerization and oligomerization of a single-ligand-bound EGFR dimer after single ligand-induced autophosphorylation of the receptor dimer (37, 55). This is consistent with the homodimeric structure of DDM-solubilized EGFR *in vitro* as described above. These results indicate that binding of only one EGF molecule induces autophosphorylation of the receptor dimer and recruits Shc1 to the phosphorylated receptor on the cell surface.

## Discussion

The present study shows that, unlike previously observed (18), detergent-solubilized full-length EGFR adopts a dimeric form before and after the receptor activation *in vitro*, when analyzed by gel filtration chromatography and cryo-ET. The dimeric form of the receptor *in vivo* is also supported by site-directed mutagenesis of amino acid residues involved in interaction between two protomers and by optical observation of ligand binding to its cell surface receptor. Like other RTKs (56, 57), these results indicate that most, if not all, of EGFR molecules adopt a dimeric form. Indeed, single-wave fluorescence cross-correlation spectroscopy analysis of fluorescent protein-labeled EGFR and ErbB2 expressed in Chinese hamster ovary cells shows that most, if not all, EGFR and ErbB2 adopt preformed homodimers and heterodimers *in vivo*, irrespective of the expression levels (35). Recent structural studies on the EGFR family members using cryo-electron microscopy demonstrate that the receptor complexes with its ligand in detergent micelles or lipid nanodiscs look very similar (58, 59), although unliganded receptor molecules have not been analyzed.

Based on analyses of the crystal structure and disulfide cross-linking of EGFR, in contrast, loose linkage between ligand binding and the activation of the receptor kinase has been proposed (22). When EGFR mutants with cysteine substitutions in the extracellular JM and TMD were studied, disulfide cross-linking of the receptor was observed only in the presence of EGF (22). In the absence of EGF, however, the spontaneously cross-linked receptors through a cysteine disulfide bridge could be autophosphorylated to some extents, endocytosed and degraded. To observe cross-linking of the receptors in the absence of bound ligand, therefore, endocytosis of the receptor should be prevented as previously demonstrated (30). Furthermore, the insertion of 20-40 amino acid residues into the extracellular linker region abolished the receptor capacity to bind ligand and caused autophosphorylation of the receptor in the absence of ligand (60, 61). These results indicate a significant functional linkage between the ECD and ICD through TMD in the wild-type receptor.

When EGFR was expressed at low levels,< 5 molecules per μm^2^, in *Xenopus* oocytes, the receptor was predominantly monomeric in the absence of ligand (24). This claim was based on photobleaching of stable EGFR spots in position and counted the number of steps until fluorescence intensities disappear. After incubation with 2 μM EGF for 1-2 min, however, more than 25% of spots were still photobleached with a single step. This result indicates that a significant fraction of the spots with single-step photobleaching contains dark spots due to incomplete maturation of the fluorophore. This incomplete maturation is particularly true at low temperatures, such as 18°C required for the cultivation of *Xenopus* oocytes (62). The number of dark spots is likely to be underestimated, and dimers and even oligomers might be counted as monomers.

Numerous studies have demonstrated that in the absence of bound ligand, ~40-100% EGFR molecules adopt a preformed dimeric structure, depending upon methods and cell lines used for the analyses (30, 32–37, 63). Considering inefficient fluorescent protein folding and chemical crosslinking (64, 65), the fraction of the receptor dimer is likely to be, if at all, underestimated. The inactive dimer or predimer fraction, ~40%, of total EGFR molecules expressed in NIH 3T3 cells (66) is also underestimated since the EGFR molecules form heterodimers with ErbB2 endogenously expressed in NIH 3T3 cells (37, 67). Reversible firefly luciferase enzyme fragment complementation assays show that prior to ligand binding, most, if not all, EGFR molecules adopt a preformed dimeric form (68). While EGF binding augmented luciferase activity due to dimerization of an ICD-deleted EGFR, the luciferase activity of the full-length EGFR decreased presumably due to a conformational change of the preformed dimer of the receptor (68). Reversible luciferase complementation assay also indicates that other RTKs, such as TrkA and TrkB (56, 57), adopt a preformed dimeric structure.

Prior to ligand binding, therefore, it now appears that most, if not all, of EGFR molecules adopt a preformed dimeric structure *in vitro* and *in vivo* through the interaction of the receptor ICDs, in which the AP-2 helix and “electrostatic hook” play crucial roles, as shown in the present study. The TKD (12, 14, 16), TMD (69) and extracellular subdomains II and IV (11) also seem to contribute to the formation of the unliganded dimer. All the EGFR family members spontaneously form homodimers and heterodimers in endoplasmic reticulum (ER) before reaching the cell surface (37), as observed in other RTKs (56, 57, 70, 71). The dimer spontaneously formed in ER, which is not dependent on the expression levels of the receptor (35, 55), is stable and does not dissociate. The preformed dimeric structure of EGFR may also ensure its inactive form prior to ligand binding. Random collision of monomeric EGFR molecules on the cell surface could spontaneously activate the receptor and would be harmful to the cell. EGFR activation modeling predicts that the activation of the preformed dimers would be 100-fold faster than that of the monomers (34).

The present study indicates that dimerization (tetramerization) and oligomerization of the receptor dimer occur after phosphorylation of the receptor upon ligand binding (**Figure 5C**; **Supplementary Figures S11D, E**), as previously observed (37). Consistently, homo-FRET analysis by Hofman et al. (55) also demonstrated that EGF binding did not cause dimerization or oligomerization of the receptor at detectable levels *in vivo*, when a kinase-dead (K721A) EGFR mutant or an EGFR mutant where nine tyrosine residues as major phosphorylation sites were replaced with phenylalanine. Therefore, tetramerization and oligomerization of EGFR previously observed (33) occurs after phosphorylation of the receptor upon ligand binding.

The present study also shows that only a single ligand binding event activates EGFR *in vivo*. Liu et al. (72) also reported the similar result based on Western blot analysis of co-expressed mutant EGFRs, kinase-deficient receptor and ligand-binding-deficient one that functioned as a receiver/acceptor kinase. These results are consistent with the preformed dimeric structure of EGFR and negative cooperative binding of ligand to EGFR, which is seen when the binding of a ligand to the first site on a dimer reduces ligand affinity for the second site on the dimer. Indeed, Alvarado et al. (73) determined two crystal structures of *Drosophila* EGFR extracellular domain dimers without bound ligand and with a single bound ligand as a symmetric unliganded dimer and an asymmetric, single-liganded dimer, respectively. The authors propose that a single ligand binding event to the unliganded symmetric dimer with two identical binding sites induces conformational changes that promote asymmetry in the dimer and constrains the second binding site to reduce its affinity for ligand. The receptor dimer with negative cooperative ligand binding is more sensitive to ligand at low concentrations than the monomeric receptor.

It has been shown that the C-terminal tail inhibits self-autophosphorylation by the TKD, suggesting that self-autophosphorylation of the C-terminal tail is likely a mechanism for removing inhibitory constrains on enzyme activity (74, 75). Indeed, the mutations in the AP-2 helix and the “electrostatic hook” in the present study are likely to dissociate the symmetric inactive ICD dimer, resulting in spontaneous activation of the receptor (**Figures 4B, C**). EGFR mutants with C-terminal deletions, EGFRvIVa and EGFRvVIb, have been found in human glioblastoma multiforme (41–43). These two mutants have transforming and tumorigenic properties and show ligand-independent constitutive activation. EGFRvV has the C-terminal truncation from Gly-959 and exhibits increased ligand-dependent kinase activity (41, 42). The oncogenic function of these mutants depends on their intrinsic kinase activity, and the proximal region of the C-terminal tail (residues 959 to 1030) participates in autoinhibitory interactions (45, 76). This proximal region contains the AP-2 helix and the “electrostatic hook”, which play roles in the formation of symmetric inactive TKD dimer. Therefore, the autoinhibitory function of the C-terminal tail is likely to stabilize the symmetric inactive TKD dimer. It has also been proposed that residues Tyr-992 to Leu-1014 make specific docking interactions in *cis* with the C-lobe of the activator/doner TKD and thus contribute to autoinhibition (76).

It has recently been proposed that ligand binding to the ECD of EGFR induces the conformational change of the ICD in monomeric EGFR (77). Unfortunately, however, the authors did not experimentally analyze whether the EGFR synthesized *in vitro* is monomeric or dimeric. The receptor seems to be dimeric since it was phosphorylated in the absence of bound ligand.

## Materials and methods

### Plasmid construction

A DNA fragment coding for full-length human EGFR was amplified from the plasmid pNUT/EGFR (30) as a template by PCR using a forward primer, 5’-GGGCTAGCATGCGACCCTCCGGGACG, in which the *Nhe* I site is underlined, and a reverse primer, 5’-GGCTCGAGTCATGCTCCAATAAATTCACTGCTTTG with the *Xho* I site underlined. The resulting PCR product was cloned between the *Nhe* I and *Xho* I sites of a pIRES2-ZsGreen1-Thr-His8 expression vector, a derivate of pIRES2-ZsGreen1 (Clontech) with a thrombin digestion site, LVPRGS, before the His tag. The thrombin digestion site was then replaced with a TEV protease digestion site, ENLYFQG, by inserting an In-Fusion fragment prepared with two pairs of PCR primers, 5’-CGTCAGATCCGCTAGCATGCGACCCTCCGGGA/3’-CTGGAAATAGAGGTTCTCTCCGTTTGTTGCTCCAATAAATTCACT and 5’- GAGAACCTCTATTTCCAGGGATCGGATCCGCACCATCACCACCATCACCATCAC/3’-GTTAACAACAACAATTGCATTCATTTTATGTTTCAGGTTCAGGGGGAGGTGTGGG, where the *Nhe* I and *Mfe* I restriction enzyme sites are underlined and In-Fusion sites are underlined with dots in bold, using an In-Fusion cloning kit (Takara Bio, Shiga, Japan). After digesting with *Nhe* I and *Mfe* I, the resulting fragment was replaced with the *Nhe* I-*Mfe* I fragment of pIRES2-ZsGreen1-Thr-His8, resulting in pIRES2-ZsGreen1-EGFR-TEV-His8.

cDNA encoding human Shc1was amplified by PCR from pcDNA3.1His p66Shc1 (Plasmid #32574; addgene), with a pair of oligonucleotide primers, 5’- CACCAAGCTTATGAACAAGCTGAGTGGAGGCG and 5’- AACCGCGGCAGTTTCCGCTCCACAGGTTGC, wherein the *Hin*dIII and *Sac* II sites are underlined, respectively. The resulting PCR product was cloned into a pAcGFP1-N1 vector (#632469; Clontech) digested with restriction enzymes *Hin*dIII and *Sac* II to make pAC-N1-GFP-Shc1, in which Shc1 was fused to the C-terminus of AcGFP1. To generate pAc-Grb2-GFP, human *GRB2* cDNA was amplified by PCR using a pair of oligonucleotide primers, 5’- CACCAAGCTTATGGAAGCCATCGCCAAATATG (forward) and 5’- AACCGCGGGACGTTCCGGTTCACGGGGGTG (reverse), from a cDNA library of HEK293T cells and then cloned into pAcGFP1-N1 in which human GRB2 was fused to the N-terminus of AcGFP1. To construct pIRES2-EGFR-GFP-Shc1, a cDNA fragment encoding full-length *EGFR* was amplified from pNUT-EGFR (30), and AcGFP1 fused Shc1 fragment was amplified from pAC-N1-GFP-Shc1. The amplified cDNAs encoding *EGFR* and *AcGFP1-Shc1* were transferred to upstream and downstream of the internal ribosome entry site (IRES) sequence of pIRES2-ZsGreen (Clontech), respectively. To construct pIRES2-EGFR-Grb2-AcGFP1, a DNA fragment encoding *Grb2-AcGFP1* was amplified from pAc-Grb2-AcGFP1 and then replaced the AcGFP1-Shc1fragment of IRES of pIRES2-EGFR-AcGFP1-Shc1. Similarly, full-length ErbB4 was amplified by PCR from pBiFC-ErbB4-JMa-VN (37), using a pair of oligonucleotide primers, 5’-CGTCAGATCCGCTAGCATGAAGCCGGCGACAGGACTTTG and 5’-GAAGCTTGAGCTCGAGTCACACCACAGTATTCCGG. Using In-Fusion, the resulting PCR product was inserted into pIRES2-EGFR-Grb2-AcGFP1 after removing its EGFR fragment by digesting with *Nhe* I and *Xho* I.

To construct pcDNA3.1-EGFR, full-length *EGFR* was amplified from pIRES2-EGFR-AcGFP1-Shc1 and transferred to pcDNA3.1 (Invitrogen). Two double mutations, F973R/L977R and E981R/D982K, were introduced to pcDNA3.1-EGFR by In-Fusion using oligonucleotide primers encoding the mutations, 5’-CCAACCGGTACCGTGCCCGGATGGATGAAG/5’-CACGGTACCGGTTGGAGTCTGTAG and 5’-GATGAACGAAAGATGGACGACGTGGTGGATGCCGAC/5’-CATCTTTCGTTCATCCATCAGGGCACGGTAGAAGTT, wherein the mutation sites are underlined, respectively. To construct pIRES2-EGFR(K721A)-AcGFP1-Shc1, a mutation, K721A, was introduced to pIRES2-EGFR-AcGFP1-Shc1 by In-Fusion using oligonucleotide primers, 5’-CGCTATCGCAGAATTAAGAGAAGCAAC and 5’-AATTCTGCGATAGCGACGGGAATTTTAAC, in which the mutation sites are underlined.

### Protein expression and purification

Full-length EGFR was expressed by transforming HEK293T cells (American Type Culture Collection) in a 15-cm Petri dish with a mixture of 20 µg pIRES2-ZsGreen1-EGFR-TEV-His8 and polyethylenimine (PEI; Polysciences, Warrington, PA) at a 1:3 (w/w) ratio of DNA to PEI. HEK293T cells were cultured in Dulbecco’s modified Eagle medium (DMEM; Gibco) supplemented with 10% (v/v) fetal bovine serum (FBS; Invitrogen) and 2 mM glutamine. This culture medium was replaced with FBS-free DMEM, 5 h prior to transformation. Cells derived from 24 dishes (15 cm in diameter) were harvested 48 h after the transformation and washed once with PBS (pH 7.4). Approximately 9 × 10^8^ cells freshly harvested, or cells stored at -80°C were incubated for 3 h in 20 ml of bursting buffer, consisting of 20 mM HEPES (pH 7.4), 0.1 mM EDTA, 0.1 mM EGTA, 5.0 mM MnCl_2_, and an EDTA-free protease inhibitor cocktail (1.0×; Nacalai Tesque, Kyoto, Japan). Cells were disrupted with a Dounce homogenizer on ice and centrifuged at 50,000 ×*g* in a micro ultracentrifuge (model CS150GXL; Hitachi Koki, Tokyo, Japan) for 30 min at 4°C. Pellets were suspended in 20 ml of solubilization buffer, consisting of bursting buffer supplemented with 1.0% (w/v) DDM (Thermo Fisher Scientific) or 1.0% (w/v) Triton X-100 (Nacalai Tesque) for 3 h and were cleared by centrifugation at 150,000 ×*g* for 30 min at 4°C. The supernatant was then mixed with 1.0 ml of Ni Sepharose resin (GE Healthcare), which was equilibrated with solubilization buffer, on an orbital shaker for 1.0 h in a cold room. The suspension was then collected in a Poly-Prep affinity chromatography column (9 cm in height, 10 ml reservoir volume, and 2 ml bed volume; Bio-Rad) by gravity and washed with 20-times bed volume of washing buffer (20 mM Tris-HCl, pH 8.0; 400 mM NaCl; and 0.01% DDM) supplemented with 20 mM imidazole. The column was then washed with the same volume of washing buffer supplemented with 30 mM imidazole, followed by washing with the same volume of washing buffer supplemented with 40 mM imidazole. EGFR was eluted with five-times the bed volume of washing buffer supplemented with 250 mM imidazole, and the eluate was fractionated into five fractions of 1.0 ml each. To determine fractions containing EGFR, 15 μl of each fraction was analyzed by PAGE using precast 10% Extra PAGE gels (Nacalai Tesque). Collected fractions, which contained 2-4 μM EGFR (~1.5 ml in total), were dialyzed overnight against 1.0 liter of dialysis buffer (20 mM Tris-HCl, pH 8.0; 200 mM NaCl; and 0.01% DDM).

Purified full-length EGFR, 15 μl, was mixed with the same volume of 2 x Laemmli sample buffer (Bio-Rad) containing 5% (v/v) β-mercaptoethanol and then heated at 95°C for 5 min. The samples were separated by using precast 10% Extra PAGE gel with running buffer (25 mM Tris-HCl, pH 8.6; 192 mM glycine; 0.1% (w/v) SDS) at room temperature. Proteins on the gel were fixed in solution [40% (v/v) methanol and 10% (v/v) acetic acid in water] for 15 min at room temperature and then stained with 0.25% (w/v) Coomassie Brilliant Blue G-250 (Nacalai Tesque) in acidic methanol [45% (v/v) methanol and 10% (v/v) acetic acid in water] for 30 min at room temperature. The gel was destained with aqueous 10% (v/v) acetic acid until visible bands appeared.

### Gel electrophoresis and phosphorylation assay

In PAGE analysis (**Supplementary Figure S1**), purified EGFR (10 μg in 15 μl of dialysis buffer) was subjected to SDS-PAGE analysis as described above. For Western blotting after gel electrophoresis, proteins on the gel were transferred to polyvinylidene difluoride (PVDF) membrane (pore size, 0.45 μm; GE Healthcare) using a TurboBlot dry blotting system (Bio-Rad) and were then immunostained with antibodies, D-8 (sc-365829, Santa Cruz Biotechnology) for EGFR and pY-20 (sc-508, Santa Cruz Biotechnology) for phosphorylated EGFR. Horse radish peroxidase (HRP)-conjugated anti-mouse IgG antibody (GE Healthcare) was used as a secondary antibody for the detection of EGFR bound with the first antibody.

For the phosphorylation assay (**Supplementary Figure S2**), an aliquot (15 μl of 20 μg/ml) of purified EGFR was reacted at 30°C for 15 min with or without 100 ng EGF in the presence of 1.0 mM ATP in phosphorylation buffer containing 25 mM HEPES (pH 7.4), 20 mM MgCl_2_, 5 mM β-glycerophosphate, 0.5 mM DTT, and 0.1 mM NaVO_3_. The reaction was stopped by adding the same volume of two-fold concentrated Laemmli sample buffer and was analyzed by SDS-PAGE and Western blotting as described above.

For the autophosphorylation analysis (**Figures 4B, C**) of doubly mutated EGFRs, F973R/L977R and E981R/D982K, plasmid constructs, pcDNA3.1-EGFR, pcDNA3.1-EGFR(F973R/L977R) and pcDNA3.1-EGFR(E981R/D982K), were transfected to the modified HeLa cells, which were seeded in a Coster^®^ 6-well plate (Corning, NY) at a density of 1 x 10^5^ cells/well in growth media (DMEM supplemented with 10% (v/v) FBS) one day before transfection. Next day, the media in the wells were replaced with 2 ml of fresh media. A transfection mixture, containing 1.5 μg plasmid DNA and 3 μg PEI in 200 μl Opti-MEM (Gibco), was incubated at room temperature for 10 min and was then added to each well. The plate was incubated for 4 h at 37°C in a 5% CO_2_ atmosphere. After the incubation, media in wells were replaced with 2 ml of fresh growth media, and the plate was incubated at 37°C in a 5% CO_2_ atmosphere. After incubation for 26 h, wells were washed three times with 2 ml of fresh growth media and were covered with 2 ml of Dulbecco’s MEM without FBS for serum starvation, followed by incubation at 37°C for 14 h in a 5% CO_2_ atmosphere. After starvation, the plate was placed on ice for 10 min, and washed twice with 2 ml of ice-cold Dulbecco’s PBS. Cells in each well were lysed by adding 70 μl Laemmli buffer containing 5% (v/v) β-mercaptoethanol, 1.0 mM Na_3_VO_4_, a phosphatase inhibitor cocktail (PhosSTOP; Sigma) and a protease inhibitor cocktail (cOmplete EDTA free; Sigma). An aliquot, 15 μg of total proteins, of the lysed cells was incubated at 95°C for 7 min and was subjected to SDS-PAGE analysis, which was performed using 7% (w/v) acrylamide gels in running buffer [25 mM Tris-HCl (pH 8.6), 192 mM glycine, 0.1% (w/v) SDS]. Proteins on the gel were transferred to PVDF by electrophoresis using a Mini Trans-Blot cell (Bio-Rad) at 30 V for 16 h in a cold room. The membrane was probed with a primary antibody, mouse anti-phosphotyrosine monoclonal (pY-20, 1/500 dilution; Santa Cruz Biotechnology), rabbit anti-EGFR monoclonal (D38B1, 1/3000 dilution; Cell Signaling Technology), or rabbit anti-phosphotyrosine (pY1173) monoclonal (53A5; 1/1000 dilution; Cell Signaling Technology), and then with secondary antibodies, HRP-conjugated anti-mouse IgG (dilution, 1/3000; GE Healthcare) and HRP-conjugated anti-rabbit IgG (dilution, 1/3000; GE Healthcare), respectively. Phosphorylation signals were detected using ECL prime (Amersham Biosciences) and recorded by LAS-3000 imager (FujiFilm, Tokyo, Japan). Phosphorylation intensity was quantified using ImageJ. This autophosphorylation analysis was repeated five times and a representative result is shown in **Figure 4B**.

### Gel filtration chromatography

Full-length EGFR, 2 µM at a final concentration, in dialysis buffer was reacted with or without 20 µM EGF (recombinant human; Abbiotec, Escondido, CA) for 30 min on ice in the presence of 1.0 mM ATP in 1.0 ml of total reaction volume, and an aliquot (500 µl) was analyzed by gel filtration column chromatography at a flow rate of 0.4 ml/min (24 ml bed volume, 10 mm inner diameter, 300 mm in height; prepacked with Superose 6 Increase; GE Healthcare), which was equilibrated with running buffer (20 mM Tris-HCl, pH 8.0; 200 mM NaCl; and 0.01% DDM), by fast protein liquid chromatography (FPLC) using an AKTA-Explorer (GE Healthcare) in a cold room.

Purified EGFR-TEV-His8 (0.15 mg) was digested with 0.05 mg (250 unit) of TEV protease (Accelagen, San Diego, CA) in 0.6 ml of buffer, which contains 20 mM Tris-HCl (pH 8.0), 200 mM NaCl, and 0.01% DDM, for 18 h at 4°C. An aliquot (500 μl) was subjected to analysis by gel filtration chromatography using a column prepacked with Superose 6 Increase 10/300 GL as described above. A representative result from three independent experiments is shown in **Figure 1B**.

Apparent Stokes radii of full-length EGFR in the presence or absence of bound EGF were determined using the following proteins as standard markers in gel filtration chromatography: bovine thyroid thyroglobulin (Mw, 669 kDa; Stokes radius, 8.5 nm; GE Healthcare), horse spleen ferritin (440 kDa, 6.1 nm; GE Healthcare), and rabbit muscle aldolase (158 kDa, 4.8 nm; GE Healthcare).

### Cryo-ET

Colloidal gold particles (10 nm; Amersham Biosciences), 2 µl of a “1.0 OD” solution, were equilibrated with purification buffer (20 mM Tris-HCl, pH 8.0; 200 mM NaCl; and 0.01% DDM). Purified EGFR (20 µl, 0.2 mg/ml) before or after incubation with 20 μM EGF at a final concentration on ice for 30 min was mixed with the equilibrated colloidal gold particles at 3:1 ratio for alignment purposes. A Quantifoil holey carbon copper grid (R 1.2/1.3; Electron Microscopy Sciences, Hatfield, PA) was glow-discharged for 60 s, and the EGFR and colloidal gold mixture (3 μl) was spotted on the grid. The grid was blotted with filter paper (grade 595; Ted Pella, Redding, CA) for 3 s and was vitrified at 4°C with 80%-90% humidity (78) using a Vitrobot Mark IV plunge-freezing device (FEI). An FEI Titan Krios equipped with a Falcon II direct electron detector was operated at 300 kV (accelerating voltage) and at a magnification of 37,000 with the defocus value of -2.0 μm for data collection (resulting in a pixel size of 2.258 on the specimen scale). Specimens were tilted from 0° to –70° and 0° to +70° with an increment of 1° tilt/image. The total dose for each tilt series did not exceed 90 e^-^/Å^2^ to minimize radiation damage. Tomography software (version 4.0; Thermo Fisher Scientific) was used for data acquisition.

### Image processing and docking

Unliganded and liganded EGFR tilt-series were aligned using 10-nm gold particles as fiducial markers with mean errors of 3.5 Å (1.55 pixels) and 3.7 Å (1.6 pixels), respectively. A series of 2D slices perpendicular to the tilt axis were reconstructed by using a radius-weighted back-projection algorithm from a stack of extracted areas from the tilt images. This stack of 2D slices constituted the initial 3D map and was also called a back-projection map or a tomogram. The back-projections were run on multiple selected areas, generating volumes of 800 × 800 × 800 voxels. The tomograms were further improved *via* a regularizing process by COMET (47, 79, 80), version 6.4.2, which enhances the contrast of density to increase the signal-to-noise in the final tomograms. The MINER program of the package was used to identify coordinates for desired maps in the regularized tomograms using either a 3D voxel or a molecular mass range as a parameter. From the list of coordinates, an automated extraction of subtomograms was performed in a volume of 110 × 110 × 110 voxels that were already low-pass filtered to 15 Å. Individual snapshots of the extracted subtomograms were generated using BOB software (81) with a volume-rendering option. The CORRPAIR program of the package was applied to the individual 3D subtomograms to create a correlation matrix pairing all the subtomograms. The correlation matrix contained clusters of subtomograms that had similar correlations. The CORRAVE program of the package was then run to maximize the correlations within each cluster and to generate an averaged subtomogram from each cluster. CHIMERA (48), version 1.14.0 (http://www.cgl.ucsf.edu/chimera/), was subsequently used for docking the model of EGFR domains from PDB entries to the averaged subtomograms of the clusters. The crystal structures of EGFR domains were aligned to the individual subtomogram maps by using CHIMERA with the manual option. The PDB entries used for docking were 1NQL (11), 3NJP (22), 2M0B (82), 2M20 (62), 3GT8 (16), and 2GS6 (14). These representative clusters were cross-correlated with the 20 Å-resolution low-pass filtered crystal structures of EGFR domains. We used all the crystal and NMR structures available for docking and the structures docked to the envelopes with the highest correlation coefficient were chosen. In **Figures 3A, B**, 1NQL was docked to C1, C2, C3, C6, C8, C10, C12, C13, C15, C16, C18, C19, C22, C23, C24, C25; half of 3NJP to C4, C5, C7, C9, C11, C14, C17, C20, C21; 2M0B to all TMD of unliganded EGFR; 3GT8 to all TKD of unliganded EGFR; 3NJP to all ECD of liganded EGFR; 2M20 to all TMD of liganded EGFR; and 2GS6 to all TKD of liganded EGFR.

### Electron density distribution along the axis of minimum moment of inertia

Electron density maps were represented by a set of vectors (*x_i_*, *y_i_*, *z_i_*, and *d_i_*) (1 ≤ *i* ≤ N), where *x_i_*, *y_i_*, and *z_i_* are the coordinates of each voxel, and *d_i_* is the density of each voxel. N is the number of voxels in each density map. The coordinates (*x_i_*, *y_i_*, *z_i_*) of each density map were transformed to fit the center of mass of the density map to the origin of the coordinate system (*x’_i_*, *y’_i_*, *z’_i_*), using equation (1).


(1) $$\left(x_{i}^{'},\;y_{i},\;z_{i}^{'}\right)=\left(x_{i}\;-\frac{\sum_{i}x_{i}d_{i}}{\sum_{i}d_{i}},\;y_{i}-\frac{\sum_{i}y_{i}d_{i}}{\sum_{i}d_{i}},\;z_{i}-\frac{\sum_{i}z_{i}d_{i}}{\sum_{i}d_{i}}\right)\;$$

The inertia tensor I of the electron density map was calculated using equation (2).


(2) $$\text{I}=\left[\begin{array}{lll} \Sigma_{i}d_{i}({y^{\prime }}_{i}^{2}+{z^{\prime }}_{i}^{2}) & -\Sigma_{i}d_{i}{x^{\prime }}_{i}{y^{\prime }}_{i} & -\Sigma_{i}d_{i}{x^{\prime }}_{i}{z^{\prime }}_{i}\\ -\Sigma_{i}d_{i}{y^{\prime }}_{i}{x^{\prime }}_{i} & \Sigma_{i}d_{i}({z^{\prime }}_{i}^{2}+{x^{\prime }}_{i}^{2}) & -\Sigma_{i}d_{i}{y^{\prime }}_{i}{z^{\prime }}_{i}\\ -\Sigma_{i}d_{i}{z^{\prime }}_{i}{x^{\prime }}_{i} & -\Sigma_{i}d_{i}{z^{\prime }}_{i}{y^{\prime }}_{i} & \Sigma_{i}d_{i}({x^{\prime }}_{i}^{2}+{y^{\prime }}_{i}^{2}) \end{array}\right]$$

The axis of minimum or maximum moment of inertia was determined from an eigenvector that give a minimum or maximum eigenvalue, λ, respectively, in the following equation:


I·n=λn

where n is a 3D unit vector. The coordinates of each density map, (*x’_i_*, *y’_i_*, *z’_i_*), were rotated around the center of mass so that the eigenvectors of the minimum and maximum moments of inertia can be aligned to the z- and x-axes, respectively. Among the two possible opposite alignments along the z-axis, one of the two alignments was chosen based on the docked models of crystal structures (**Figures 3A, B**). The data processing above was performed on R (https://www.r-project.org/) with custom scripts.

Each aligned density map was sectioned into two voxels (~4.5 Å) each along the z-axis, and the electron density within each section was plotted against the z-axis. The mean values of the plots of 25 unliganded or 25 liganded EGFR density maps are shown in **Figure 2C**, after normalized by total volumes. Error bars indicate standard deviation (SD). Asterisks indicate significant differences (*p< 0.05, **p< 0.01) of the two groups with two-sided Student’s *t*-test (in the range between -140 Å and 75 Å), or Mann-Whitney U-test (in the range over 80 Å) using R.

### HeLa cells lacking EGFR family members

HeLa cells (RIKEN BRC, Saitama, Japan) were cultured in DMEM supplemented with 10% (v/v) FBS and incubated in a humidified incubator containing 5% CO_2_ at 37°C. To knockout EGFR family members in HeLa cells, a Cas9-RNA complex transfection system (Alt-R CRISPR-Cas9; Integrated DNA Technologies, Coralville, IA) was used. A custom-made guide RNA was complexed with Cas9 protein, and the resulting RNA and protein complex was transfected to HeLa cells with Lipofectamine RNAiMAX (Thermo Fisher Scientific) according to the manufacturer’s instructions. After three days, the transfected cells were transferred to 96-well plates for the isolation of single cells. The isolated cells were further cultured for 1–2 weeks, and their genomes were analyzed by PCR for deletion, which was then confirmed by DNA sequencing. To knockout of multiple EGFR family members, the above knockout procedure was repeated three times to create a triple-knockout cell line, “*124* KO HeLa”, which lacks EGFR, ErbB2, and ErbB4. The target 20-nucleotide genome sequences of guide RNA for EGFR, ErbB2, and ErbB4 are 5’-AGGGTTGTTGCTGAACCGCA in exon #4, 5’-TGAGTCCATGCCCAATCCCG in exon #7, and 5’-TGCTGCCATCGAGAATGTGC in exon #6, respectively. All genome deletions introduced to the cell line created stop codons within the extracellular domain regions of each receptor. As ErbB3 is not expressed on the HeLa cell surface (95), we used a HeLa cell line lacking EGFR, ErbB2, and ErbB4, which was confirmed by Western blot analysis and ligand binding optically observed described below (**Supplementary Figure S3**).

### Optical observation

To label EGF with fluorescent dyes, 1.0 mg/ml EGF (PeproTech, Cranbury, NJ) solution in water was mixed with the same volume of 100 mM potassium phosphate buffer, pH 7.0. Alexa Fluor 555 NHS (Thermo Fisher Scientific) or Alexa Fluor 647 NHS in dimethyl sulfoxide was added to the EGF solution to a final concentration of 400 μM. The reaction mixture was incubated at room temperature for 70 min and then loaded onto a PD-10 desalting column (GE Healthcare) to remove unbound dye molecules. Eluates from the column were concentrated with a centrifugal filter device (Amicon Ultra 3K; Millipore). Concentrations of EGF and fluorescent dyes in the concentrated samples were determined using a spectrophotometer (NanoDrop; Thermo Fisher Scientific) based on molecular extinction coefficients, 18000 M^-1^cm^-1^ at 280 nm for EGF, 150000 M^-1^cm^-1^ at 550 nm for Alexa555, and 239000 M^-1^cm^-1^ at 650 nm for Alexa647. We used only EGF samples with higher labelling efficiency than 95%.

To prepare a trolox and troloxquinone mixture (TXTQ), 2.5 mg/ml trolox (Cayman Chemical, Ann Arbor, MI) was dissolved in 10 mM potassium phosphate, pH 7.2. After dissolving, the pH of the solution was adjusted to pH 7.0 using 1.0 M NaOH. Approximately 10% of trolox (~0.25 mg/ml) was converted to troloxquinone by oxidization under illumination using a mercury lump (Olympus) on a stereo microscope (SZX16; Olympus). Generation of troloxquinone was monitored by measuring the absorbance at 255 nm using a spectrophotometer. Oxidization was continued until the absorbance at 255 nm reached ~1.2 in 0.1 mm path length (83).

To construct cell lines co-expressing EGFR and GFP-tagged Shc1 or EGFR and GFP-tagged Grb2, a plasmid construct, pIRES2-EGFR-AcGFP1-Shc1, pIRES2-EGFR-Grb2-AcGFP1, or pIRES2-EGFR(K721A)-AcGFP1-Shc1, was transfected into the modified HeLa cell line that did not express EGFR, ErbB2, ErbB3, or ErbB4 on the cell surface. Cells (0.5 × 10^3^) were seeded in a glass-bottom dish (35 mm in diameter; Iwaki, Shizuoka, Japan) one day before transfection, and the next day, medium was replaced with 0.3 ml of fresh growth medium, DMEM supplemented with 10% (v/v) FBS. Transfection mixture, which contained 0.3 μg plasmid DNA and 0.6 μg of PEI in 30 μl of Opti-MEM (Gibco), was incubated at room temperature for 10 min and was then added to each dish. The dish was incubated at 37°C for 4 h under a 5% CO_2_ atmosphere. After incubation, growth media in the wells were replaced with fresh media, and the plates were further incubated at 37°C for 40 h in a 5% CO_2_ atmosphere. Then, the dish was washed three times with 0.3 ml of DMEM without FBS and filled with 0.3 ml of DMEM without FBS for serum starvation. The dish was further incubated at 37°C for more than 3 h in a 5% CO_2_ atmosphere.

Optical observation of EGF binding to the cell surface of the modified HeLa cell was performed using an inverted microscope (Eclipse Ti; Nikon) with an oil-immersion objective (SR APO TIRF ×100/1.49; Nikon) at room temperature. GFP, Alexa555, and Alexa647 were excited by a laser unit (LU-N4; Nikon) with 488 nm, 561 nm, and 640 nm, respectively. The fluorescent signal was split into three EM CCD cameras (DU-897; Andor Technology, Belfast, UK) using dichroic mirrors (FF580-FDi01 and FF662-FDi01; Semrock, Rochester, NY) and bandpass filters (FF01-525/45, FF01-600/37, and FF01-692/40; Semrock). Before observation, cells were washed twice with Hank’s balanced salt solution (HBSS; Gibco) and covered with 200 μl HBSS containing 1.0 mM TXTQ. The apical surface of the cell was observed under oblique illumination (54). At 10 s after video recording started, 200 μl HBSS containing 1.0 mM TXTQ, 0.5 mg/ml glucose oxidase, 0.04 mg/ml catalase, 1.0 mg/ml glucose, and fluorescently labeled EGF was applied to the dish. As shown in **Supplementary Figure S12**, the fluorescently labeled EGF activated EGFR at the similar level to that by unlabeled ligand. Movements of fluorescent spots derived from fluorescently labeled EGF, GFP-Shc1 or Grb2-GFP on the cell surface were recorded at 10 frames/s for 90 s after EGF stimulation of the cell.

The images of three-color channels were shifted and distorted, primarily due to chromatic aberration. We used an ImageJ plugin (DoM_Utrecht, Netherlands) to correct the aberration. The GFP and Alexa647 channels were corrected to fit the Alexa555 channel. Fluorescent bead images of a calibration slide (Tool for calibration Multi Spec #1783-455; Zeiss) were used as references for correction. After correction, fluorescent spots in each channel were detected by an ImageJ plugin (MosaicSuite; MOSAIC Group, Towson, MD) and analyzed using a custom R script. We defined colocalization of spots in different channels when the distance between the spots was less than $\sqrt{2}$ pixel (0.21 μm). The fluorescent spots in different channels sometimes approached each other within the threshold distance by chance without actual colocalization. The number of this “pseudo-colocalization” in different channels was estimated by the colocalization analysis, where one of two images was flipped vertically and horizontally. The mean of two numbers of colocalization with vertically and horizontally flipped images was used as the number of the pseudo-colocalization for every frame and subtracted from the number of colocalization of the original unmodified two images to obtain the corrected numbers of colocalization shown in **Figure 5** and **Supplementary Figure S11**, for every pair of different channel images. In case of the number of colocalizations of all three channels, a colocalized image of Alexa555–EGF and Alexa647–EGF was used for the colocalization analysis with the remaining Shc1 (or Grb2) channel image in the same way.

### Statistical analysis

Statistical analysis of data was performed using R (version 3.6.3) or SigmaPlot (version 13.0). All data were checked for normality of distribution and homogeneity of variance using χ<σπ>2</σπ> goodness of fit test (*p*< 0.05) and were evaluated using two-sided Student’s *t*-test for comparisons between pairs of groups. If normality did not hold, Mann-Whitney U test or Levine’s test was used. Results are reported as mean ± SD or standard error of the mean (SEM). Asterisks in figures indicate significant differences (*p < 0.05, **p < 0.01, ***p < 0.001) of two groups.

## Data availability statement

The datasets presented in this study can be found in online repositories. The names of the repository/repositories and accession number(s) can be found below: https://www.ebi.ac.uk/pdbe/emdb/, 30714-30721 https://www.ebi.ac.uk/pdbe/emdb/, 30723-30734 https://www.ebi.ac.uk/pdbe/emdb/, 30736-30765 https://www.ebi.ac.uk/pdbe/emdb/, 30862 https://www.ebi.ac.uk/pdbe/emdb/, 30864.

## Author contributions

Conceptualization, IM.; methodology, EP, E-IS, RA, and L-GÖ.; software, GW and L-GÖ; investigation, EP, E-IS, and L-GÖ; formal analysis, EP, E-IS, L-GÖ, and IM; writing - original draft, EP, E-IS, and IM; writing – review & editing, EP, E-IS, IM, and US; funding acquisition, IM and US; supervision, IM and US. All authors contributed to the article and approved the submitted version.

## Funding

This work was supported by Okinawa Institute of Science and Technology Graduate University.

## Acknowledgments

We are grateful to T. Sassa for his construction of pAC-N1-GFP-Shc1 and pAc-Grb2-GFP, A. Mugo for his advice on purification of the full-length EGFR, and T. Murayama and H. Iha for comments on the manuscript.

## Conflict of interest

The authors declare that the research was conducted in the absence of any commercial or financial relationships that could be construed as a potential conflict of interest.

## Publisher’s note

All claims expressed in this article are solely those of the authors and do not necessarily represent those of their affiliated organizations, or those of the publisher, the editors and the reviewers. Any product that may be evaluated in this article, or claim that may be made by its manufacturer, is not guaranteed or endorsed by the publisher.
