# Supplementary material for: Allosteric activation of preformed EGF receptor dimers by a single ligand binding event
Source: Front Endocrinol (Lausanne). 2022 Nov 30;13:1042787. doi: 10.3389/fendo.2022.1042787 (PMC9748436; doi:10.3389/fendo.2022.1042787)
Supplement: Supplementary file 13 [file Table_1.docx]

| **Supplementary Table S1. Summary of cryo-ET data acquisition and image processing** | | |  |
| --- | --- | --- | --- |
|  |  |  |  |
|  |  |  |  |
| **Acquisition setup** |  |  |  |
|  | Microscope | Titan Krios |  |
|  | Voltage (keV) | 300 |  |
|  | Detector | FEI FALCON II |  |
|  | Energy-filter | No |  |
|  | Å/pixel | 2.258 |  |
|  | Magnification | 37000× |  |
|  | Acquisition scheme | Bi-directional |  |
| **Processing** | **Unliganded EGFR** | **Liganded EGFR** |  |
| # of tomogram | 18 | 15 |  |
| # of subtomogram | 474 | 557 |  |
| Defocus | –2.0 μm | –2.0 μm |  |
| Total dose (e^-^/Å^2^) | 90 | 90 |  |
| Tilt range | –70°to 70° | –70°to 70° |  |
| # of tilts for reconstruction | 134 | 135 |  |

Note: Only a single defocus was set for all the tilt series. A series of tilt angles were at a 1.0^o^ increment. Coupled core-signalling unit and Fourier cell correlation have not been applied.
